# Abnormal Expression of Connexin43 in Cardiac Injury Induced by S-Band and X-Band Microwave Exposure in Rats

**DOI:** 10.1155/2021/3985697

**Published:** 2021-12-17

**Authors:** Yue Yin, Xinping Xu, Yabing Gao, Juan Wang, Binwei Yao, Li Zhao, Haoyu Wang, Hui Wang, Ji Dong, Jing Zhang, Ruiyun Peng

**Affiliations:** Beijing Institute of Radiation Medicine, Beijing 100850, China

## Abstract

Although the effects of microwave exposure on the heart have gradually become the focus of domestic and foreign scholars, the biological effects caused by different doses and different frequency bands of exposure are still unclear. In this study, we will investigate the damaging effect of S-band and X-band microwave composite exposure on cardiac structure and function, as well as the pathophysiological significance of Cx43 in cardiac conduction dysfunction after exposure. We used S- and X-band radiation sources with the average power density of 5 and 10 mW/cm^2^ to expose Wistar rats to single or composite exposure. At the 6^th^ hour, on the 7^th^, 14^th^, and 28^th^ days after exposure, ECG was used to detect the electrical conduction of the heart, and the myocardial enzyme was measured by the automatic biochemical analyzer. We selected the observation time points and groups with severe damage to observe the changes of myocardial structure and ultrastructure with an optical microscope and TEM; and to detect the expression and distribution of Cx43 by western blotting and immunohistochemistry. After exposure, the heart rate increased, the P wave amplitude decreased, and the R wave amplitude increased; the content of the myocardial enzyme in serum increased; the structure and ultrastructure of cardiac tissue were damaged. The damage was dose-dependent and frequency-dependent. The expression of Cx43 in myocardial tissue decreased, and distribution was abnormal. Taken together, these findings suggested that the mechanism of abnormal electrical conduction in the heart of rats by S- and X-band microwave exposure might be related to the decreased expression and disordered distribution of Cx43 after microwave exposure.

## 1. Introduction

The influence of electromagnetic fields, especially microwave radiation, on biological systems has attracted much interest for a long time [1, 2]. Microwave radiation may cause different biological effects on the cardiovascular system [3, 4]. Xu et al. [5] have found that after 11 mW/cm^2^ microwave radiation in rhesus monkeys, arrhythmia occurred. ECG results indicated the decrease of the R-H value and T wave peak, and some animals had wide QRS waves, which suggested the damaged heart conduction tissues and myocardial fibers. Zhang et al. [6] observed that after long-term microwave radiation with average power densities of 0, 2.5, 5, and 10 mW/cm^2^ in rats. The myocardial enzymes activities and ion concentrations were imbalanced, which indicated microwave radiation could cause damage to rats' myocardia. Another study used radiofrequency electromagnetic radiation with a frequency of 1800 MHz to continuously radiate rats for 5 weeks. They found irregular myocardial fibers and widened fiber gaps, which might cause abnormal contraction of myocardia [7].

Cx43, a member of the gap junction protein family, is widely distributed in the cardiovascular system [8], nervous system [9], immune system [10], and tumor tissues [11], participating in normal cell-to-cell communication and mediating tumor immune response. It is the most widely distributed and most abundant protein in cardiomyocytes [12]. Cx43 plays a decisive role in the electrical conduction of the heart coexpressed with ion channels [13, 14] and leads to an increased susceptibility to arrhythmias by dephosphorylation [15].

Although a previous study addressed that the S- and X-band microwave radiation could damage the structure and the function of the heart [16], there has been no research showing the effect of S- and X-band microwave composite exposure on cardiac injury. And the relationship between heart injury and Cx43 after microwave exposure has not been reported yet. In this study, we analyzed the changes in the structure and function as well as the changes in Cx43 expression and distribution of the heart after composite exposure. Furthermore, we explored the pathophysiological significance of Cx43 in cardiac electrical conduction abnormalities after composite exposure in rats. Our research would provide novel approaches to injury mechanisms and the necessary basis of protective targets.

## 2. Materials and Methods

### 2.1. Animals and Experimental Approval

140 secondary male Wistar rats (200 ± 20 g) were purchased from Beijing Weitonglihua Experimental Animal Center. Rats were fed in an SPF-grade animal facility with the standard laboratory conditions (22 ± 1°C, 60% feeding density, 12 hours circadian rhythm, and 5 rats per cage), eating and drinking freely. All protocols were approved by the Beijing Institute of Radiation Medicine Animal Care and Use Committee. The experiments were approved by the Ethical Committee of the Academy of Military Medical Sciences, and the authorization number was IACUC-DWZX-2021-650.

### 2.2. Groups and Microwave Exposure

In this experiment, “S” and “X” represented the 2.856 GHz and 9.375 GHz microwave exposure groups. To eliminate the influence of rats' weight in the experiment, 140 rats were divided into 7 groups equally by the stratified random method. The group assignment was as follows: control group (C group), 5 mW/cm^2^ S-band exposure group (S5 group), 10 mW/cm^2^ S-band exposure group (S10 group), 5 mW/cm^2^ X-band exposure group (X5 group), 10 mW/cm^2^ X-band exposure group (X10 group), 5 mW/cm^2^ composite exposure group (SX5 group), and 10 mW/cm^2^ composite exposure group (SX10 group).

Rats were fixed in the radiation box made of plexiglass, and the rats were exposed to the radiation source with the exposure table rotated to eliminate positional influence. Rats in the single-band exposure groups were radiated for 6 min, and those in the composite-band exposure groups were radiated by each band for 6 min. During the exposure period, rats in the C group were also handled parallel to those in the exposure groups, except for microwave radiation. Before and immediately after microwave exposure, 10 rats were selected randomly from each group, and detected the core temperature by the portable thermometer JM222 (Beijing Zhongwang, China).

### 2.3. ECG Examination

At the 6^th^ hour and on the 7^th^, 14^th^, and 28^th^ days after exposure, the multichannel physiological recording and analysis system (BIOPAC, USA) was used to detect the rats' ECG. There were 5 rats in each group. And the rats were weighed and injected intraperitoneally with 1% pentobarbital sodium (1 mL/100 g). After anesthesia, the operation process was as follows: shaving the rats' limbs, fixing them in the supine position, and disinfecting with 75% ethyl alcohol. The electrode wires included red (right upper limb), black (right lower limb), and green (left lower limb). The electrode needles were, respectively, pierced into the subcutaneous of their corresponding limbs, connected to the ECG bioamplifier with a sensitivity of 2000 Hz. The rats were in a quiet state and recorded continuously for 3 min. Then we analyzed and calculated changes in indicators such as the heart rate, P wave, and R wave.

### 2.4. Serum Biochemical Analysis

At the 6^th^ hour and on the 7^th^, 14^th^, and 28^th^ days after exposure, 5 rats in each group were taken. After the rats were anesthetized (the specific operation is the same as method 3), 3 mL of blood was collected from the inferior vena cava to prepare serum. An automatic biochemical analyzer (Coulter JTIR, USA) was used to determine the activities of AST, CK, and LDH.

### 2.5. Structure of Rats' Myocardial Tissue

On the 7^th^ day after exposure, the rats were anesthetized and the hearts were removed, with 5 rats in each group. Removed hearts were fixed in 10% formalin fixative for 1 week. After dehydration, transparency, and dipping in wax, the paraffin-embedded heart tissues were cut into slices of 5 *μ*m. After dried at 60°C for 2 days and deparaffinized, the tissues were stained by H&E. The stained slices were observed and photographed by the optical microscope (Leica, Germany).

### 2.6. Ultrastructure of Rats' Myocardial Tissue

On the 7^th^ day after exposure, the rats were anesthetized and the hearts were removed, with 3 rats in each group. A fresh tissue block of about 1 mm^3^ was taken from the apex of the heart and placed into 2.5% glutaraldehyde fixative for 2 h. Next, the tissue pieces were fixed in 1% osmic acid for 1 h. After dehydration with gradient ethanol, acetone transition, and embedded in Epon 812 resin, the tissues were cut into semithin sections to locate where the damage was the most severe. Then, the semithin slices were made into 70 nm ultrathin sections and stained with lead-uranium. At last, the slices were observed and photographed by a TEM (Hitachi, Japan).

### 2.7. Expression of Cx43 in Rats' Myocardia

On the 7^th^ day after exposure, the total protein of myocardial tissues of rats was extracted and subjected to western blotting in groups C, S10, X10, and SX10. The operation steps were as follows: cut out 20 mg of each frozen heart kept in the refrigerator, added 4°C precooled PBS, washed the blood, and discarded the liquid; added 200 *μ*L tissue lysate solution at the ratio of 1 mg/10 *μ*L (lysate : protease inhibitor = 100 : 1) and cut the tissue into pieces; 3000 r/s homogenized for 4 times, 15 s each time by an automatic homogenizer, bathed in ice for 10 min, centrifuged 12000 r/min, for 15 min at 4°C, and collected the supernatant; and determined the protein concentration by the BCA protein concentration determination kit (Thermo, USA). The protein was mixed with the loading buffer (4 : 1), denatured in boiled water bath for 10 min, and added the cooled sample on the SDS-PAGE. After the steps of electrophoresis, membrane transfer, and blocking, the rabbit anti-Cx43 (TBST diluted 1 : 1000; Abcam, USA) and mouse anti-GAPDH (TBST diluted 1 : 10000; Abcam, USA) were added, shook slowly overnight at 4°C in a shaker, and washed with TBST for 3 × 10 min; the HRP-labeled goat anti-rabbit IgG (TBST dilution 1 : 10000; Beyotime, China) and goat anti-mouse IgG (TBST solution dilution 1 : 10000; Beyotime, China) were added, shook for 1 h at room temperature, and washed with TBST for 3 × 10 min; the clear bands were semiquantified the optical density value and performed statistical analysis.

### 2.8. Distribution of Cx43 in Rats' Myocardia

On the 7^th^ day after exposure, the hearts of 5 rats in groups C, S10, X10, and SX10 were fixed in 10% formalin fixative solution for 1 week, and after dehydration, transparency, wax immersion, paraffin embedding, and 5 *μ*m film production, the immunohistochemical experiment was carried out. The main steps were as follows: deparaffinized to water; put the tissue section into the boiling citrate buffer, continuously heated in a microwave oven for 10-15 min, and cooled to room temperature naturally; added the endogenous peroxidase blocker dropwise and incubated in the dark for 10 min at room temperature; added rabbit anti-Cx43 (diluted in PBS, 1 : 1000; Abcam, USA) and incubated at 37°C for 1 h; added reaction enhancement solution dropwise and incubated at room temperature for 20 min; dropped HRP-labeled goat anti-rabbit IgG and incubated at 37°C for 30 min; added DAB and observed under the microscope to control the color development time; used hematoxylin counterstain; and dehydrated transparent mount and observed and took pictures under the optical microscope. PBS was used to wash for 3 × 5 min between every two steps.

### 2.9. Statistical Analysis

Data in the article were expressed in terms of mean ± SD, and the SPSS19.0 version was used for statistical analysis. One-way ANOVA and independent sample *t*-tests were applied to statistics analysis.

The acceptable significance level for all tests was *P* < 0.05. According to the comparison objective and meaning, the significant signs were classified as follows: significant exposure effect, ^∗^*P* < 0.05 and ^∗∗^*P* < 0.01 (comparison between the radiation group and the C group); significant frequency-dependent effect, ^▵^*P* < 0.05 and ^▵▵^*P* < 0.01 (X10 and S10, X5 and S5); significant dose-dependent effect, ^#^*P* < 0.05 and ^##^*P* < 0.01 (S10 and S5, X10 and X5); and significant composite exposure effect, ^^^*P* < 0.05 and ^^^^*P* < 0.01 (SX10 and S10, SX5 and S5) and `*P* < 0.05 and ^“^*P* < 0.01 (SX10 and X10, SX5 and X5).

## 3. Results

### 3.1. Microwave Radiation Induced Abnormal ECG Results

**Figure 1:**
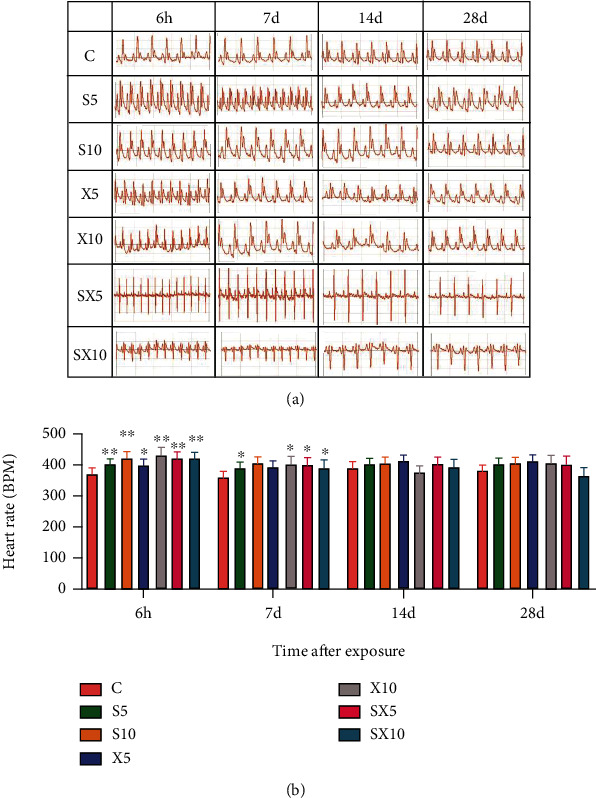
Heart rate of ECG of rats after S- and X-band microwave exposure. (a) ECG records. (b) Analysis of the heart rate. Data was expressed as means ± SD. Compared with the C group, ∗ meant *P* < 0.05 and ∗∗ meant *P* < 0.01 (*n* = 5).

**Figure 2:**
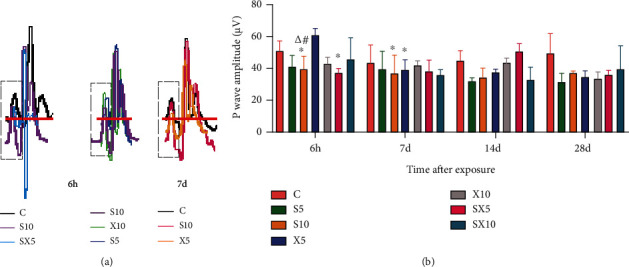
P wave of ECG of rats after S- and X-band microwave exposure. (a) Changes of the P wave (the dotted line boxes show the P wave). (b) Analysis of the P wave amplitude. Data was expressed as means ± SD. Compared with the C group, ∗ meant *P* < 0.05 and ∗∗ meant *P* < 0.01; for significant frequency-dependent effect, ▵ meant *P* < 0.05; for significant dose-dependent effect, # meant *P* < 0.05 (*n* = 5).

**Figure 3:**
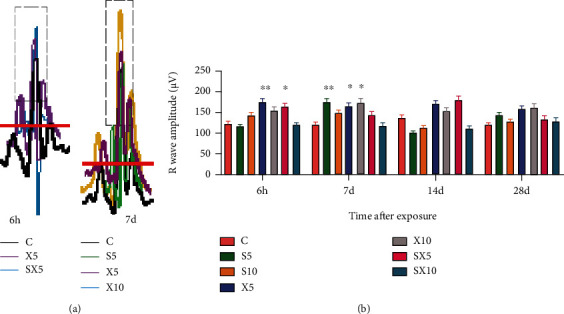
R wave of ECG of rats after S- and X-band microwave exposure. (a) Changes of the R wave (the dotted line boxes show the R wave). (b) Analysis of the R wave amplitude. Data was expressed as means ± SD. Compared with the C group, ∗ meant *P* < 0.05 and ∗∗ meant *P* < 0.01 (*n* = 5).

The heart rate, P waves, and R waves of rats were measured at the 6^th^ hour, on the 7^th^, 14^th^, and 28^th^ days after exposure (*n* = 5):

1. At the 6^th^ hour after exposure, compared with group C, the heart rate of rats increased in the radiation groups (*P* < 0.01 or 0.05). On the 7^th^ day after exposure, the heart rate still increased in the S5, X10, SX5, and SX10 groups (*P* < 0.05) (Figure 1)
2. At the 6^th^ hour after exposure, compared with group C, the P wave amplitude of the S10 and SX5 groups decreased (*P* < 0.05); the frequency-dependent effect results showed that the S10 group decreased more significantly than the X10 group (*P* < 0.05); the dose-dependent effect results stated that the S10 group decreased more significantly than the S5 group (*P* < 0.05). On the 7^th^ day after exposure, the comparison with group C suggested that the P wave amplitude of the S10 and X5 groups decreased (*P* < 0.05) (Figure 2)
3. At the 6^th^ hour after exposure, compared with group C, the R wave amplitude increased in the X5 and SX5 groups (*P* < 0.01 or 0.05). On the 7^th^ day after exposure, the R wave amplitude of the S5, X5, and X10 groups increased (*P* < 0.01 or 0.05) (Figure 3)

### 3.2. Microwave Radiation Increased Myocardial Enzyme Activity

**Figure 4:**
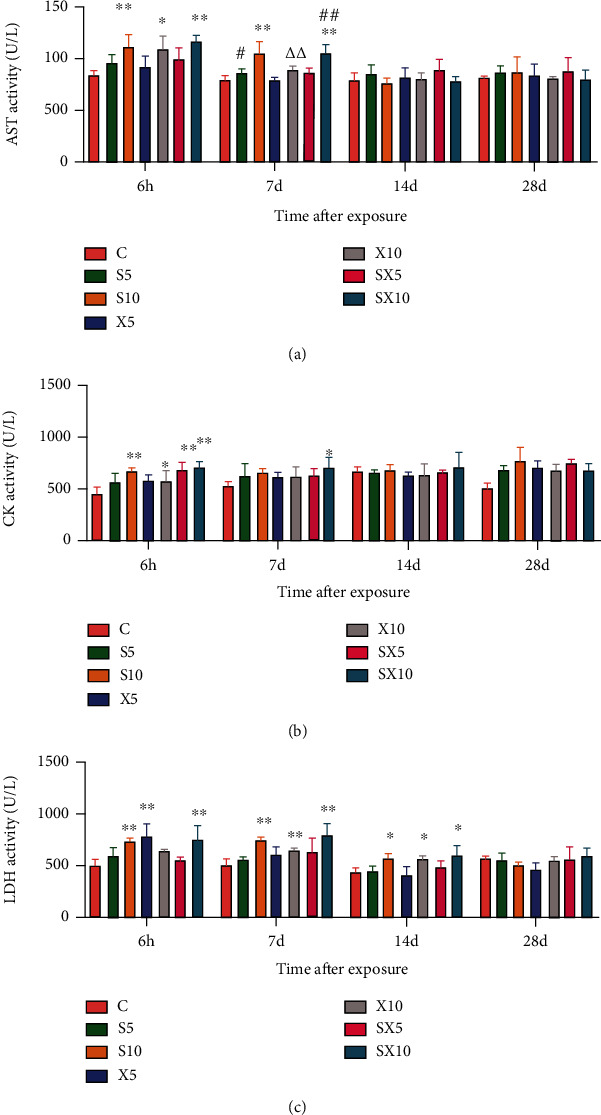
Changes of myocardial enzyme activity of rats after S- and X-band microwave exposure. (a) AST activity. (b) CK activity. (c) LDH activity. Data was expressed as means ± SD. Compared with the C group, ∗ meant *P* < 0.05 and ∗∗ meant *P* < 0.01; for significant frequency-dependent effect, ▵▵ meant *P* < 0.01; for significant dose-dependent effect, ## meant *P* < 0.01 (*n* = 5).

The activities of AST, CK, and LDH (U/L) were detected in the serum of rats at the 6^th^ hour, on the 7^th^, 14^th^, and 28^th^ days after exposure (*n* = 5):

1. At the 6^th^ hour after exposure, compared with group C, the activity of AST increased significantly in the S10, SX10, and X10 groups (*P* < 0.01 or 0.05). On the 7^th^ day after exposure, compared with group C, the activity of AST significantly increased in the S10 and SX10 groups (*P* < 0.01); and the dose-dependent effect results showed that the S10 group increased more significantly than the S5 group (*P* < 0.05), and the SX10 group increased more significantly than the SX5 group (*P* < 0.01); and the frequency-dependent effect results showed that the S10 group increased more significantly than the X10 group (*P* < 0.01) (Figure 4(a))
2. At the 6^th^ hour after exposure, compared with group C, the activity of CK increased in the S10, SX5, SX10, and X10 groups (*P* < 0.01 or 0.05); and on the 7^th^ day after exposure, the SX10 group increased (*P* < 0.05) (Figure 4(b))
3. At the 6^th^ hour after exposure, compared with group C, the activity of LDH increased significantly in the S10, X5, and SX10 groups (*P* < 0.01); and on the 7^th^ day after exposure, the S10, X10, and SX10 groups increased significantly (*P* < 0.01); on the 14^th^ day after exposure, the S10, X10, and SX10 groups increased (*P* < 0.05) (Figure 4(c))

### 3.3. Microwave Radiation Caused Abnormal Myocardial Structure

Based on the previous experiment results, at the 6^th^ hour and on the 7^th^ day after exposure, rats received heavier damage than at other time nodes. Therefore, this experiment selected the 7^th^ day with heavier damage as the time point of tissue structure observation.

**Figure 5:**
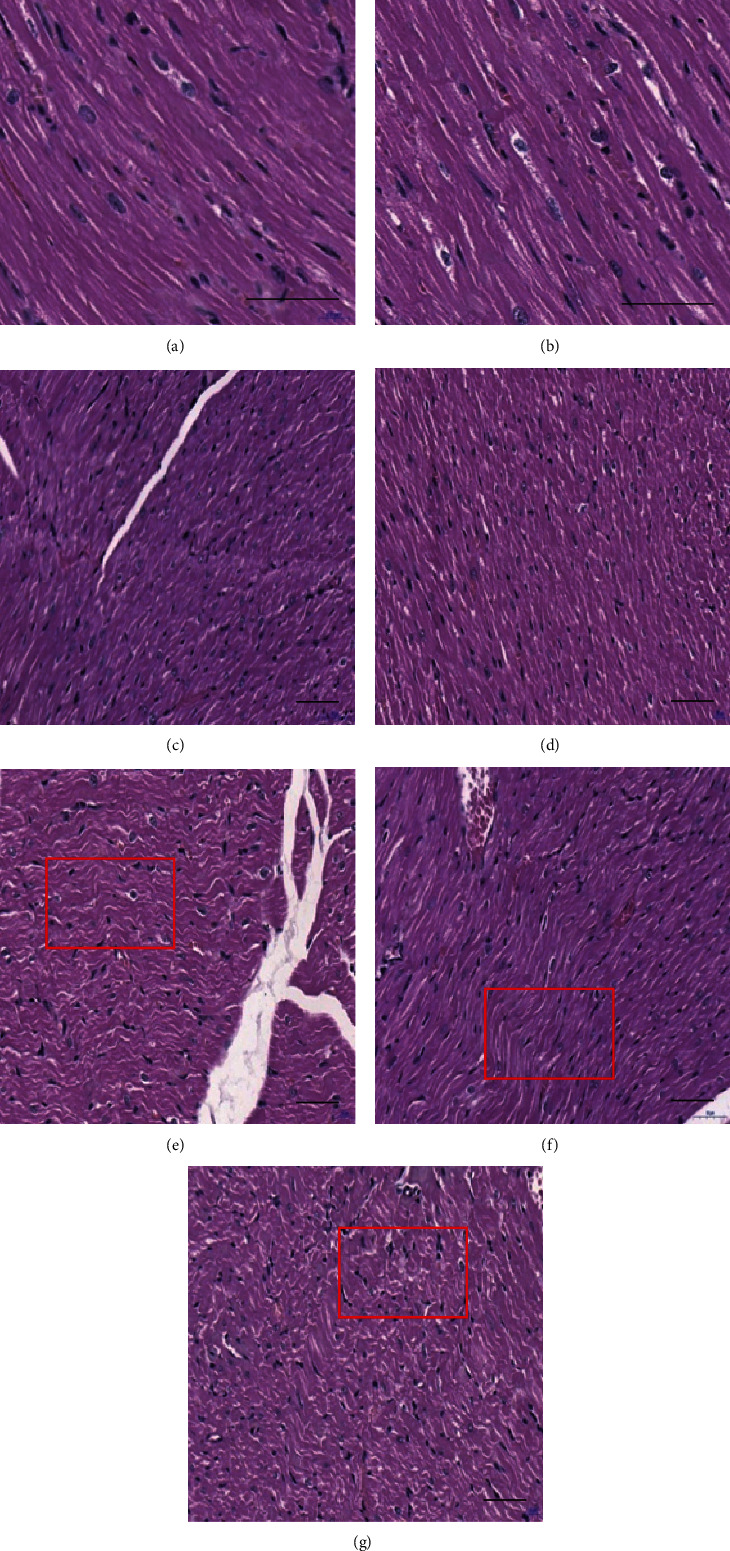
Changes of myocardial structure of rats on the 7^th^ day after S- and X-band microwave exposure. (a) C group. (b) S5 group. (c) S10 group. (d) X5 group. (e) X10 group. (f) SX5 group. (g) SX10 group. (a, b) ×400. (c–g) ×200. Scale bar = 50 *μ*m. The red frame indicated wavy fibers.

On the 7^th^ day after exposure, the myocardial fibers were neatly arranged, and nuclei of endothelial cells were fusiform, and myocardial cells were oval in the C group. In the S5, X5, and X10 groups, there was no significant change in the myocardial tissue structure. In the S10, SX5, and SX10 groups, the myocardial fiber arrangement of rats was disordered and wavy, and nuclear pyknosis was occasionally seen. The above injuries had a dose-dependent effect. The arrangement of myocardial fibers was disordered, and the structure changes after exposure, which suggested that it might be related to impaired heart function (Figure 5).

### 3.4. Microwave Radiation Caused Abnormal Myocardial Ultrastructure

**Figure 6:**
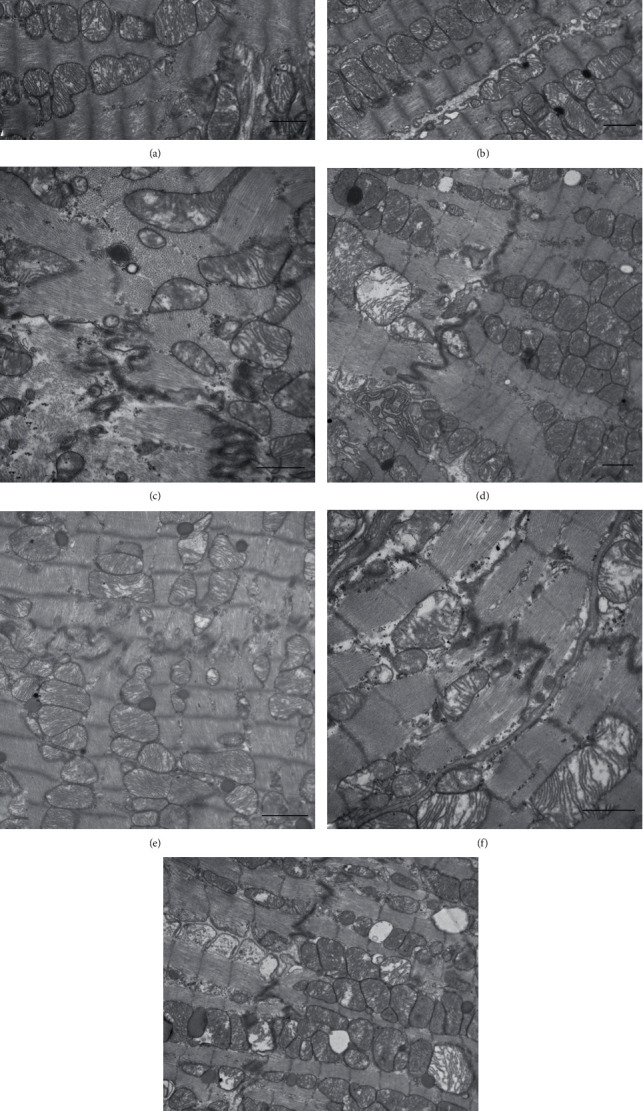
Changes of myocardial ultrastructure of rats on the 7^th^ day after S- and X-band microwave exposure. (a) C group (×15000). (b) S5 group (×10000). (c) S10 group (×20000). (d) X5 group (×12000). (e) X10 group (×12000). (f) SX5 group (×20000). (g) SX10 group (×12000). Scale bar = 1 *μ*m.

Based on the results of cardiac function testing, this experiment chose the 7^th^ day with heavier damage as the time node for tissue ultrastructure observation. On the 7^th^ day after exposure, under the electron microscope, myocardial fibers in group C were arranged neatly, mitochondrial cristae were clear and complete, and the intercalated disk structure was continuous and folded. There was no obvious abnormality in the ultrastructure of the S5 and X5 groups, and the myocardial fibers were arranged neatly, and the mitochondrial structure was relatively complete. Mitochondrial cavitation occurred in the S10, X10, SX5, and SX10 groups, intercalary disk structure was broken and incomplete, and changes in the arrangement of myocardial fibers were disordered. Mitochondrial structure damage after exposure indicated abnormal energy metabolism. Intercalated disk structure changes might cause damage to cell connection structure, so that chemical coupling and electrical coupling between cells are affected (Figure 6).

### 3.5. Microwave Radiation Decreased the Expression of Cx43 in Myocardia

**Figure 7:**
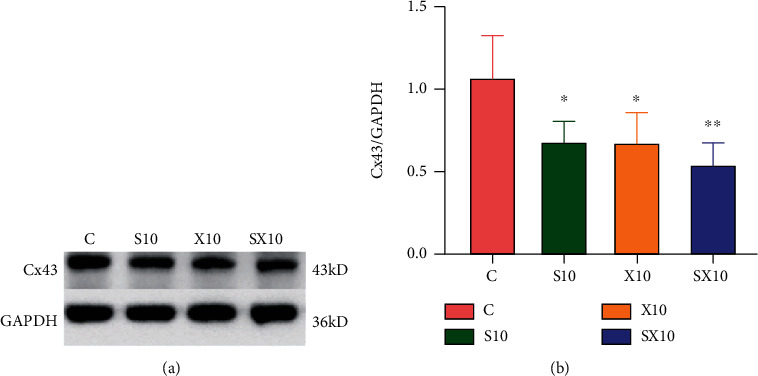
Changes of Cx43 expression in rats' myocardial tissue on the 7^th^ day after microwave radiation. (a) Cx43 protein bands. (b) Western blot quantitative analysis results.

From the above experimental results, it was found that the 10 mW/cm^2^ single and composite exposure groups suffered significant damage on the 7^th^ day after exposure. The 7^th^ day was selected as the observation time point to detect the Cx43 content in the myocardial tissues of the C, S10, X10, and SX10 groups. The total protein was extracted from myocardial tissues and western blot experiments were performed to semiquantify the content of Cx43. The results showed that on the 7^th^ day after exposure, the content of Cx43 in the SX10 group was significantly reduced compared with that in the C group (*P* < 0.01), and the content in the S10 and X10 groups was reduced (*P* < 0.05). Based on the important regulatory role of Cx43 in cardiac electrical conduction, the decrease of Cx43 suggested that it might be related to cardiac electrical conduction disorders (Figure 7).

### 3.6. Microwave Radiation Induced the Abnormal Distribution of Cx43 in Myocardia

At present, there is no reliable quantitative method for the positive signal of Cx43 immunohistochemistry, so only the optical microscope observation has been used for preliminary exploration in this experiment.

**Figure 8:**
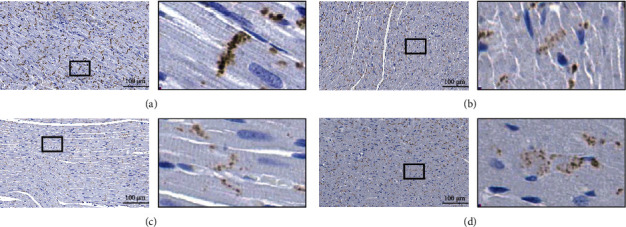
Changes of Cx43 distribution in rats' myocardial tissue on the 7^th^ day after microwave radiation. (a) C group. (b) S10 group. (c) X10 group. (d) SX10 group. (a–d) ×400. Scale bar = 100 *μ*m.

The myocardial tissues of rats in the C, S10, X10, and SX10 groups were selected for immunohistochemical experiments on the 7^th^ day after exposure. The results suggested that Cx43 in group C was distributed at the end-to-end junction of the two cells [17], in a strip shape, perpendicular to the long axis of the cardiomyocytes. Compared with the C group, the Cx43 distribution in the SX10 group was the most disordered, and the positive signal at the end-to-end junction was significantly reduced and scattered. S10 and X10 groups were reduced, but there was no significant difference between the groups. The distribution of Cx43 and the decrease of the positive signal after exposure might relate to cardiac electrical conduction disorder, which was consistent with the results of the western blot experiment (Figure 8).

## 4. Discussion

The heart is one of the target organs that are relatively sensitive to microwave radiation. Previous studies have shown that microwave radiation affected both the function and the structure of the heart. However, the effect of S- and X-band microwave composite radiation on heart damage has not been reported yet.

In the ECG waveform, the P wave is the depolarization wave of the left and right atria, and the QRS wave is the depolarization wave of the left and right ventricles [18]. Through the analysis of the waveform, we could judge the changes of depolarization potential and time in various parts of the heart, which would be helpful to diagnose and prevent arrhythmia in time. In this study, the changes of cardiac function in rats after microwave exposure were indicated by analyzing the results of ECG, and the damage of cardiac conduction function in rats at the 6^th^ hour and on the 7^th^ day after microwave exposure was confirmed, which showed that the heart rate increased, the P wave amplitude decreased, and the R wave amplitude increased. The results might be related to the changes of depolarization potential and depolarization time of atria and ventricles.

Myocardial enzymes are specific enzymes found in cardiomyocytes, including AST, CK, and LDH. When myocardial cells were damaged, myocardial enzymes would increase at a certain time, which could be combined with ECG or other relevant tests to determine the extent and type of damage [19]. In this study, the changes of cardiac function in rats after microwave exposure were detected by serum biochemical analysis. The results showed that the heart was damaged at the 6^th^ hour and on the 7^th^ day after exposure, and the activities of AST, LDH, and CK were increased. Those results mentioned above might be related to the damage of cell membrane structure and the change of permeability, which might lead to the myocardial enzyme entering the blood.

In this study, the changes in the structure and ultrastructure of the rats' heart after microwave radiation were detected by the methods of optical microscope observation after H&E staining and TEM observation. It was clear that the heart structure was damaged at the 6^th^ hour and on the 7^th^ day after exposure, and the damage was more serious on the 7^th^ day. After the 7^th^ day, a recovery trend presented. Under the optical microscope, the main manifestations of the damage showed that the myocardial structure was disordered and wavy. Under the TEM, the myocardial fiber was disordered, the mitochondria was cavitated, and the intercalary disk structure was incomplete.

These results suggested that the S- and X-band microwave radiation could cause damage to the structure and function of the rat hearts. The functional damage was severe at the 6^th^ hour and on the 7^th^ day, and the structural damage appeared on the 7^th^ day. However, 7 days later, both structural damage and functional damage were recovered. The dose- and frequency-dependent effects were consistent with the results of previous studies.

Cx43 is the most abundant gap junction protein in the heart and plays an important role in cardiac electrical conduction. Changes in its expression, distribution, and dysfunction could lead to abnormal electrical coupling between cells which could cause arrhythmia [20]. Cx43 participated not only in the formation of gap junctions between cells but also in the formation of L-type calcium channel molecular complexes [21], the regulation of outward potassium currents [22], and the composition of sodium channels [23]. Li et al. [24] radiated SD rats with electromagnetic pulses with a field strength of 200 kV/m, a pulse frequency of 200 times, and a pulse interval of 2 seconds. Based on the research, the expression of Cx43 decreased in the heart. Nav1.5 was the *α* subunit of the sodium channel in heart tissue and was the main channel for Na^+^ flow [25]. Another study found that the reduction or absence of Cx43 expression could cause the shortening of the action potential time course, the expression of Nav1.5, and the reduction of sodium current mediated by it [26]. It was suggested that the downregulation of Cx43 and Nav1.5 increased the susceptibility of conduction disorders and arrhythmia [27]. Cx43 has also been shown to be widely present in immune cells [28, 29], which could regulate lymphocyte activation and inflammatory cytokine production [30].

The damage to the electrical conduction function of the heart after microwave radiation had been confirmed, but the damage mechanism had not been elucidated. In order to explore the mechanism of injury, this study focused on the role of Cx43 in the damage of cardiac electrical conduction after microwave composite exposure. By using immunohistochemistry, western blotting, and image analysis techniques to study the changes of Cx43 in rats' myocardial tissue after microwave composite exposure. The Cx43 expression clearly decreased, and its distribution was disordered after microwave radiation of 10 mW/cm^2^; also, the SX10 group suffered the most serious damage. The S10 and X10 groups were severely injured, but there was no difference between the two groups. The above results suggested that the decrease of Cx43 expression and abnormal distribution might lead to abnormal electrical conduction in the heart, but how to analyze the relationship between Cx43 and abnormal electrical conduction requires further study.

This research has potential limitations. Due to the inconsistent installation conditions of the microwave radiation sources in various laboratories, there is a lack of repeatability in the radiation conditions and the method of establishing the model. In addition, due to the characteristics of microwaves, it is necessary to use nonmetallic materials while carrying out biological effect experiments, which greatly limits the collection of some real-time data. The abovementioned problems are also common problems in this field, and there is an urgent need to introduce scientific research instruments with the function of shielding electromagnetic signals.

## 5. Conclusion

In conclusion, abnormal heart function and myocardial structure damage in rats after exposure suggested that the heart injury model after S- and X-band microwave radiation was successfully established. Based on this model, the research also provided a possible mechanism for the abnormal electrical conduction of the heart after exposure; that is, the decreased and abnormal distribution of Cx43 after exposure could cause damage to the electrical conduction function of the heart. This experiment provided a new direction for the exploration of the abnormal electrical conduction mechanism of the heart after microwave radiation and provided a new intervention target for the study of damage prevention methods.

## Data Availability

The data used to support the findings of this study are included within the article.

## Abbreviations

Cx43: Connexin43
SPF: Specific-pathogen-free
ECG: Electrocardiogram
TEM: Transmission electron microscope
AST: Aspartate aminotransferase
CK: Creatine kinase
LDH: Lactate dehydrogenase
H&E: Hematoxylin and eosin
PBS: Phosphate-buffered saline
BCA: Bicinchoninic acid
SDS-PAGE: Sodium dodecyl sulfate-polyacrylamide gel electrophoresis
TBST: Tris-buffered saline with Tween-20
GAPDH: Glyceraldehyde 3-phosphate dehydrogenase
HRP: Horseradish peroxidase
DAB: Diaminobenzidine
SD: Standard deviation
ANOVA: Analysis of variance
BPM: Beat per minute
SD: Sprague Dawley
Nav1.5: Sodium channel protein 1.5 subtype.
